# Difference in the short-term outcomes of laparoscopic gastrectomy for gastric carcinoma between the east and west: a retrospective study from the IMIGASTRIC trial

**DOI:** 10.7150/jca.31192

**Published:** 2019-07-10

**Authors:** Jian-Xian Lin, Jun-Peng Lin, Jacopo Desiderio, Jian-Wei Xie, Alessandro Gemini, Jia-bin Wang, Jun Lu, Qi-Yue Chen, Long-long Cao, Mi Lin, Ruhong Tu, Chao-Hui Zheng, Ping Li, Amilcare Parisi, Chang-Ming Huang

**Affiliations:** 1 Department of Gastric Surgery, Fujian Medical University Union Hospital, Fuzhou, Fujian Province, China; 2 Key Laboratory of Ministry of Education of Gastrointestinal Cancer, Fujian Medical University, Fuzhou, Fujian Province, China; 3 Department of Digestive Surgery, St. Mary's Hospital, University of Perugia, Terni, Italy

**Keywords:** stomach neoplasm, laparoscopic gastrectomy, surgical outcomes, postoperative complications, Clavien-Dindo classification

## Abstract

**Purpose**: To compare the clinicopathologic data and short-term surgical outcomes of laparoscopic gastrectomy (LG) for gastric cancer (GC) between the east and west.

**Methods**: Patient demographics, surgical procedures, pathological information, and postoperative recovery were compared among gastric cancer patients who underwent LG in the clinical trial of IMIGASTRIC (NCT02325453) between 2009 and 2016.

**Results**: More younger males, higher BMI, lower ASA score and less neoadjvant chemotherapy were evident in east patient cohort. Eastern patients had a higher proportion of proximal, differentiated and advanced gastric cancers. More total gastrectomies, larger extent of lymph node (LN) dissection, and higher number of retrieved LNs were found in the eastern patients. However, more Roux-en-Y anastomosis procedures during distal gastrectomy and intra-corporeal anastomosis were performed in the western patients. The west patients showed faster postoperative recovery than the eastern patients. The mortality rates of the western patients were comparable to those of the eastern patients. However, fewer III-IV complications were evident in the eastern centers. Multivariate analyses revealed that an elderly age, higher ASA score, and more blood loss were the significant independent risk factors of postoperative complications for eastern patients. However, for the western patients, the independent risk factors were neoadjuvant therapy, more retrieval LNs, and pT3-4 stage.

**Conclusions**: The selections and short-term surgical outcomes of LG for GC were widely different between East and West. To obtain more objective and accurate results, these differences should be considered in future international prospective studies.

## Introduction

Despite a global decline in incidence, there are about 2.2 million new cases of gastric cancer per year, resulting in 834 thousand deaths, making gastric cancer the second leading cause of cancer related mortality worldwide 1. Since 1989, when Dubois et al. first reported their experience with laparoscopic cholecystectomy 2, abdominal surgery has increasingly moved toward a minimally invasive approach due to the many advantages regarding improvements in patient quality of life during the postoperative period. Laparoscopic approaches for gastric cancer in particular have been slowly accepted in the west, largely due to the lower incidence of gastric cancer in these countries. Currently, laparoscopic distal, subtotal, and total gastrecomy for early and advanced gastric cancer are emerging in the west with progressive acceptance among various groups 3-5. Because several epidemiologic differences exist between east and west, the delayed acceptance of gastrectomy may be partly related to skepticism regarding the oncological efficacy of laparoscopic surgery. However, few studies have focused on the outcomes of the use of laparoscopic techniques for gastric cancer between Eastern and Western countries.

We present data from the International study group on Minimally Invasive surgery for GASTRIc Cancer (IMIGASTRIC) trial 6, 7, which is a multicenter study on gastric cancer developed to collect information on the surgical, clinical, and oncological features of patients undergoing gastrectomy with a robotic, laparoscopic, or open approach. To the best of our knowledge, this study is the first large-scale case-control study to compare the surgical outcomes of laparoscopic gastrectomy (LG) for gastric cancer between the east and west.

## Patients and Methods

### Patients

This study is a multi-institutional retrospective study that compares the short-term outcomes of LG for gastric carcinoma between eastern and western countries. Data collection started after reporting the specific study protocol. This study was registered at clinical trials.gov with a registration number of NCT02325453. This study was approved by the ethics Committee of CEAS Umbria. Tailored Web-based software was developed to standardize information, facilitate the process of data collection in a unified multi-institutional database, and guarantee the proper storage of patient's data. The purpose was to create an international registry with a high methodological quality. In the present study, the data regarding procedures performed up to the data collection (from January 2009 to August 2016) that entered into the IMIGASTRIC registry were analyzed. All involved centers were referral institutions with well-established gastric cancer programs and experience in minimally invasive surgery. All diagnostic and surgical interventions at these centers were performed according to international guidelines and the information stored in institutional prospective data collection systems. The inclusion criteria were as follows: gastric cancer reported in the biopsy report; upper endoscopy and CT scan for staging; either early or advanced gastric cancer; and curative surgery. The exclusion criteria were as follows: evidence of metastatic disease, a history of another surgery for gastric cancer, emergency surgery with bleeding or perforation, another malignancy, and surgery with palliative intent. Finally, 1858 eastern patients (from three centers) and 253 western patients (from ten centers) were included in the study. The surgical procedures were described in detail as follows: 1) total or subtotal gastrectomy was performed, according to the tumor location, size, and depth of invasion, and 2) D1+/D2 lymphadenectomy was undertaken according to the rules of the Japanese Research Society for Gastric Cancer 8, 9. Tumor staging was performed according to the 8th edition of the International Union against Cancer (UICC) TNM classification 10.

Patients were observed for 30 days following surgery, and short-term surgical outcomes including the operation time, estimated blood loss, postoperative complications, length of postoperative hospital stay, number of dissected lymph nodes (LNs) and clinicopathological characteristics were recorded in a web-based system (https://imigastric.logix-software.it/). Then, the data were extracted using pre-established IT tools. Postoperative complications were classified according to the revised version of the Clavien-Dindo classification system suggested by Dindo et al. 11, 12. Types of postoperative complications were classified in accordance with the Japan Clinical Oncology Group Postoperative Complication Criteria according to the Clavien-Dindo classification ver. 2.0 13. The operation time was measured from the first skin incision to the closure of all skin incisions with skin staples. The mortality rate was defined as death within 30 days or in-hospital mortality when admitted beyond 30 days.

### Statistical analysis

All statistical analyses were performed using SPSS v18.0 for Windows (SPSS Inc., Chicago, IL). The data are presented as the mean ± standard deviation for continuous variables and as a number for categorical variables. For non-parametric data, variables were reported as the median (interquartile range). The differences in the patient demographics, surgical variables and pathological variables between the groups were calculated by using Fisher's exact test, the *t-*test, or the chi square test as appropriate. To evaluate the predictive factors for postoperative morbidity, a multivariate analysis was performed with binary logistic multiple regression analysis using dummy variables. P values less than 0.05 were considered statistically significant.

## Results

### Patient clinicopathological characteristics

The clinicopathological characteristics of the 2111 patients (1858 eastern vs. 253 western) are listed in Table 1. The western cohort comprised more elderly patients, more female patients, and patients with a higher body mass index (BMI) and a higher ASA score than the eastern cohort (all P<0.05). The proportion of patients with previous surgery and neoadjuvant chemotherapy was significantly higher in the western cohort than in the eastern cohort (both P<0.05). However, no significant differences in comorbidities were evident between the two cohorts (P>0.05).

There were more eastern patients than western patients had upper 1/3 tumors (31.9% in the east vs. 15.4% in the west; P<0.05). The eastern patients had a higher percentage of differentiated tumors with higher T stage and N stage compared to Western patients (all P<0.05). The median tumor size was similar between the cohorts (P>0.05).

### Surgical characteristics

The type of resection, type of reconstruction and anastomosis approach were significantly different between the cohorts (all P<0.05). The number of conversions to open surgeries, number of intraoperative blood transfusions, operation time, and volume of estimated blood loss were significantly lower in the eastern cohort than in the western cohort (all P<0.05). The number of patients who underwent D2 lymphadenectomy and R0 resection was significantly higher in the eastern cohort than in the western cohort (both P<0.05). The number of received lymph nodes was higher in the east than in the west (33.5±13.1 vs. 21.2±12.6, P<0.05) (Table 2).

### Perioperative outcomes

The proportion of postoperative transfusions was significantly lower in the east than in the west (1.7% vs. 9.9%, P<0.05). However, the time to NG tube removal, time to first flatus, time to start of a liquid diet, time to start of a soft diet, and the post-operative hospital stay were significantly shorter in the west than in the east (all P<0.001, Table 2).

One or more complications occurred in 277 patients (14.9%) in the east and 40 patients (15.8%) in the west, and no statistical significance was evident (P>0.05). Anastomotic stenosis (0.8% vs. 0.1%, p=0.040) and urinary system complications (2.0% vs. 0.4%, p<0.001) were more frequent in the west than in the east. However, no significant differences were evident for the other complications between the two cohorts (P>0.05).

According to the Clavien-Dindo classification, more postoperative major complications (grade III-IV) occurred in the west than in the east (5.5% vs. 2.8%, P<0.05), but for grade I-II or grade V complications, no significant differences were evident between the two cohorts (both P>0.05). Among the grade I-II complications, intra-abdominal bleeding (P=0.031), pleural effusion (P=0.002), and urinary system complications (P=0.003) occurred more frequently in the west than in the east, but the most frequent in the east were associated with pneumonia (P=0.010). Among the grade III-IV complications, more western patients than eastern patients suffered from anastomotic leakage (2.4% vs. 0.5%, P=0.002) and intestinal obstruction (0.8% vs. 0.1%, P=0.040) after LG, but no significant differences were evident for the other complications between the two cohorts (P>0.05) (Table 3).

### Predictable risk factors for postoperative complications

 Univariate analysis and multivariate analysis were used to evaluate the factors that influenced postoperative complications. On univariate analysis, an age >75 years (P<0.001), a higher ASA score (P=0.002), more comorbidities (P=0.032), and an estimated blood loss ≥75 ml (P=0.004) were significantly related to the occurrence of a postoperative complications in the east. However, in the west, comorbidities, previous surgery, neoadjuvant therapy, the anastomosis approach, combined resection, operation time, estimated blood loss, pT stage, and pN stage were significantly related to the occurrence of a postoperative complication (all P<0.05) (Table 4).

Multivariate analysis found that an age >75 years (P<0.001), a higher ASA score (P=0.017), and an estimated blood loss ≥75 ml (P=0.004) were independent risk factors for postoperative complications in the east. However, for the western patients, the independent risk factors were neoadjuvant therapy (P=0.012), the retrieval of more LNs (P=0.042), and pT3-4 stage (P<0.05) (Table 5).

## Discussion

Currently, LG is becoming more widely used worldwide and some Eastern centers have become one of the standard surgical approaches for early gastric cancer 9. LG for advanced gastric cancer has been developed successfully in many centers, and the number ongoing multicenter RCTs that focus on the safety and efficacy of LG in the east is increasing 14-16. For the west, in 1992, Ohgami et al. reported the first laparoscopic wedge resection (LWR) for the treatment of early gastric cancer (EGC) 17.

Recently, LG has become an important surgical procedure for radical gastrectomy in many western centers. However, no reports on the efficacy of LAG between the east and west centers are available. This study is the first large case-control study using multicenter clinical research data (IMIGASTRIC trial), and can be used as a preliminary basis for future prospective studies. In this study, the 1858 patients in the east and 253 patients in the west had significant differences in demographic, clinical, and pathologic characteristics. Patient characteristics such as age, and BMI and the ASA score were higher in the west. This result may be due to epidemiological differences between the east and west 18. However, differences in the selection of laparoscopic surgery cases between the east and west may have influenced the results. Hamashima C KY et al. found that the age of population-based screening is early in eastern countries, resulting in an age of onset that is lower in the east than in the west 19. The higher BMI in the western patient group may correspond to an overall higher BMI in the western population. Regarding the pathological features, more undifferentiated earlier-stage tumors were evident in the west than in the east for laparoscopic surgery, which was consistent with the study by Strong VE et al. 20. In addition, we found that the majority of patients in both cohorts had tumors in the distal stomach, but the proportion of upper 1/3 tumors was significantly higher in the east than in the west (31.9% vs. 15.4%, P<0.05), which was contrary to previous studies 18. Laparoscopic total gastrectomy for proximal gastric cancer is a difficult procedure, and fewer western centers may choose this approach for laparoscopic surgery. However, in the east, the number of gastric cancer cases was higher, the patients were younger and had less BMI, and these factors increased the number of options for laparoscopic surgery. Recently, several studies have shown that the incidence of proximal gastric cancer in the east has increased annually 21-23, possibly explaining higher proportion Eastern patients undergoing proximal and total gastrectomy in the current study.

The number of conversion to open surgeries, number of blood transfusions, and volume of estimated blood loss were higher in the west than in the east, and the operation time was longer in the west than in the east; however, the number of LN dissections was lower in the west than in the east. These observations may be attributed to differences in the patient characteristics between the east and west. More options for the treatment of early gastric cancer exist in the west than in the east, so the extent of LN dissection is smaller. Due to several factors in the west, such as the lower overall incidence of gastric cancer and higher BMI, which can increase the difficulty of laparoscopic operation, the number of conversions to open surgery, the operation time, and the volume of estimated blood loss were higher in the west than in the east. In the east, the data have suggested a learning curve number of 20-40 procedures 24. In another report from the west, Kunisaki C et al. showed that the learning curve for surgeons comprised approximately 60 cases, after which the operation time significantly improved with equivalent LN retrievals and surgical outcomes 25. The incidence of gastric cancer is lower in the west than in the east. However, the learning curve for performing minimally invasive gastrectomy requires more cases. When we compared postoperative recovery between the west and east, we found that the time to NG tube removal, time to first flatus, time to start of a liquid diet, time to the start of a soft diet and postoperative hospital stay were significantly shorter in the west than in the east, which may be related to the early implementation and application of enhanced recovery protocols (ERAS).

The incidence of postoperative complications is still the most frequently used surrogate marker of the 'quality' of surgery. Previous reported morbidity rates for laparoscopic surgery vary from 6.1 to 25.4% 15, 26-28. In this study, the postoperative morbidity was 14.9% in the east and 15.8% in the west, without significant differences. When the complications were categorized with the Clavien-Dindo classification, we found that grade III-IV complications occurred more frequently in the west than in the east (5.5% vs. 2.8%, P<0.05), which mainly contributed to the higher incidence of anastomotic leakage and intestinal obstruction. In the west, due to early drain removal or no drain placed after surgery, when anastomotic leakage or other complications occur, more invasive procedures, such as abdominal paracentesis and drainage were performed, and lead to increases in the grade of complications. Multivariate analysis further revealed that an elderly age, a higher ASA score, and a higher estimated blood loss were the independent risk factors for postoperative complications in the east, which was consistent with several previous eastern studies 29, 30. In the west, neoadjuvant therapy, pT3-4 and the number of LN dissections were the independent risk factors for postoperative morbidity, which suggested that advanced gastric cancer and a wider range of LN dissection would increase the incidence of postoperative complications in the western centers. Haskins and Badgwell et al. reported that postoperative morbidity and mortality were not increased by neoadjuvant therapy 31, 32. However, the open and laparoscopic groups were not analyzed separately in these studies, which may have caused the results to be different from those of the present study. Most of the gastric cancer patients who underwent neoadjuvant therapy had more advanced stages and needed larger extents of LN dissection, increasing the difficulty of the operation and the likelihood of morbidity, especially for laparoscopic surgery with higher technical requirements.

In conclusion, distinct differences are evident between the east and west regarding the clinicopathological characteristics and short-outcomes of laparoscopic surgery for gastric cancer. Although no significant differences in overall complications were evident between the two cohorts, the major postoperative complications (grade III-IV) and risk factors for morbidity were significantly different. Therefore, for the prospective study of the efficacy of laparoscopic radical gastrectomy for gastric cancer between the east and west, we should recognize the objective factors and pay more attention to the surgical indications and case selections to obtain more objective and accurate results.

## Figures and Tables

**Table 1:** Patient demographics and tumor finding between the eastern and western centers

Characteristics	All patients (n=2111)		X^2^/*t*	P
	Eastern (n=1858)	Western (n=253)		
Age			154.04	<0.001
<40	65(3.5)	7(2.8)		
40-75	1607(86.5)	149(58.9)		
>75	186(10.0)	97(38.3)		
Mean(±SD)	61.0±11.2	69.4±12.8	10.92	<0.001
Sex			66.65	<0.001
Male	1407(75.7)	130(51.4)		
Female	451(24.3)	123(48.6)		
Body mass index (kg/m2)	22.2±3.2	25.9±14.1	7.24	<0.001
ASA score			1027.96	0.000
1	1139(61.3)	12(4.7)		
2	646(34.8)	57(22.5)		
3	66(3.6)	182(71.9)		
4	7(0.4)	2(0.8)		
Comorbidities			3.48	0.062
Yes	1276(68.7)	159(62.8)		
No	582(31.3)	94(37.2)		
Previous surgery			6.18	0.013
Yes	413(22.2)	74(29.2)		
No	1445(77.8)	179(70.8)		
Neoadjuvant chemotherapy			66.60	<0.001
Yes	42(2.3)	31(12.3)		
No	1803(97.0)	222(87.7)		
Tumor Location			34.30	<0.001
Upper	593(31.9)	39(15.4)		
Middle	453(24.4)	93(36.8)		
Lower	812(43.7)	121(47.8)		
Tumor size(cm)	4.6±2.7	4.8±5.7	0.253	0.800
Histologic type			16.73	<0.001
Differentiated	1308(70.4)	146(57.7)		
Undifferentiated	550(29.6)	107(42.3)		
No. metastatic LNs				
pT stage			137.91	<0.001
T1	438(23.6)	126(49.8)		
T2	200(10.8)	35(13.8)		
T3	532(28.6)	69(27.3)		
T4a	685(36.9)	18(7.1)		
T4b	3(0.2)	5(2.0)		
pN stage			73.46	<0.001
N0	655(35.3)	156(61.7)		
N1	269(14.5)	35(13.8)		
N2	307(16.5)	27(10.7)		
N3a	380(20.5)	29(11.5)		
N3b	247(13.3)	6(2.4)		
pTNM stage			110.72	<0.001
IA	365(19.6)	110(43.5)		
IB	152(8.2)	33(13.0)		
IIA	185(10.0)	30(11.9)		
IIB	206(11.1)	24(9.5)		
IIIA	189(10.2)	16(6.3)		
IIIB	299(16.1)	28(11.1)		
IIIC	462(24.9)	12(4.7)		

**Table 2:** Surgical procedures between the eastern and western centers

Characteristics	All patients (n=2111)		X^2^/*t*	P
	Eastern (n=1858)	Western (n=253)		
Type of resection			73.18	<0.001
Distal gastrectomy	811(43.6)	170(67.2)		
Total gastrectomy	1007(54.2)	69(27.3)		
Proximal gastrectomy	39(2.1)	12(2.1)		
Pylorus-preserving gastrectomy	1(0.1)	2(0.8)		
Type of reconstruction			1095.99	<0.001
B-I	682(36.7)	0(0.0)		
B-II	121(6.5)	37(14.6)		
Rou-en-Y GJ	8(0.4)	133(52.6)		
Roux-en-Y EJ	1007(54.2)	66(26.1)		
Jejunal interposition	0(0.0)	4(1.6)		
Esophagogastrostomy	39(2.1)	11(4.3)		
Gastro-gastrostomy	1(0.1)	2(0.8)		
Anastomosis approach			1019.24	<0.001
intra-corporeal	99(5.3)	203(80.2)		
extra-corporeal	1759(94.7)	50(19.8)		
Extent of LN dissection			603.87	<0.001
D1	15(0.8)	50(19.8)		
D1+	13(0.7)	55(21.7)		
D2	1830(98.5)	148(58.5)		
No. of retrieved lymph nodes	33.5±13.1	21.2±12.6	-14.06	<0.001
Residual tumor			12.54	0.002
R1	28(1.5)	12(4.7)		
R0	1830(98.5)	241(95.3)		
Conversion to open			109.60	<0.001
Yes	1(0.1)	16(6.3)		
No	1857(99.9)	237(93.7)		
Operation time (min)	179.1±53.7	217.0±84.6.2	9.71	<0.001
Estimated blood loss (ml)	74.8±101.0	129.1±153.4	6.99	<0.001
Intraoperative blood transfusion			22.43	<0.001
Yes	45(2.4)	20(7.9)		
No	1813(97.6)	233(92.1)		
Postoperative transfusions			56.425	<0.001
Yes	32(1.7)	25(9.9)		
No	1826(98.3)	228(90.1)		
NG tube removal (days)	5.1±3.0	2.8±2.8	-4.31	<0.001
Time to first flatus (days)	3.7±1.3	2.7±1.0	-6.95	<0.001
Time to start of liquid diet(days)	5.1±1.8	2.3±2.1	-13.77	<0.001
Time to start of soft diet (days)	7.9±2.9	3.3±1.9	-14.61	<0.001
Postoperative hospital stay (days)	13.1±8.1	8.1±8.2	-9.93	<0.001

**Table 3:** Postoperative complications between the eastern and western centers according to Clavien-Dindo classification

Characteristics	All patients (n=2111)		X^2^/*t*	P
	Eastern (n=1858)	Western (n=253)		
**Total complications**	277(14.9)	40(15.8)	0.142	0.706
** I-II complications**	223(12.0)	24(9.5)	1.264	0.243
Abdominal infection	28(1.5)	3(1.2)	0.159	0.690
Anastomotic leakage	21(1.1)	0	2.888	0.089
Anastomotic site bleeding	5(0.3)	3(1.2)	4.956	0.060
Anastomotic stenosis	0	1(0.4)	7.347	0.120
Delayed gastric emptying	14(0.8)	1(0.4)	0.405	0.524
Intestinal obstruction	22(1.2)	1(0.4)	1.286	0.257
Intra-abdominal bleeding	2(0.1)	2(1.3)	10.247	0.031
Chylous leakage	13(0.7)	0	1.781	0.182
Pancreatic fistula	4(0.2)	0	0.546	1.000
Pleural effusion	0	3(1.2)	22.063	0.002
Pneumonia	62(3.3)	1(0.4)	6.655	0.010
Wound infection	25(1.3)	2(0.8)	0.461	0.543
Circulatory system	4(0.2)	0	0.546	1.000
Hematological system	3(0.2)	2(0.8)	3.729	0.112
Urinary system	8(0.4)	5(2.0)	8.693	0.003
Other	12(0.6)	0	1.643	0.200
** III-IV complications**	52(2.8)	14(5.5)	5.499	0.019
Abdominal infection	2(0.1)	1(0.4)	1.298	0.255
Anastomotic leakage	10(0.5)	6(2.4)	9.950	0.002
Anastomotic site bleeding	5(0.3)	1(0.4)	0.125	0.724
Anastomotic stenosis	1(0.1)	1(0.4)	2.743	0.225
Intestinal obstruction	1(0.1)	2(0.8)	8.516	0.040
Intra-abdominal bleeding	10(0.5)	1(0.4)	0.088	0.767
Pneumonia	12(0.6)	0	1.643	0.200
Sepsis	3(0.2)	1(0.4)	0.644	0.400
Wound infection	2(0.1)	0	0.273	1.000
Circulatory system	4(0.2)	0	0.546	1.000
Disseminated intravascular	2(0.1)	0	0.273	1.000
Acute renal failure	0	1(0.4)	7.347	0.120
** V complications**	2(0.1)	2 (0.8)	5.490	0.073

**Table 4:** Univariate analysis of morbidity risk factors for eastern and western laparoscopy gastrectomy

Variable	Eastern Centers				Western Centers		
	No	Yes	P		No	Yes	P
Age			0.000				0.370
<40	63(4.0)	2(0.7)			7(3.3)	0	
40-75	1381(87.3)	226(81.6)			127(59.6)	22(55.0)	
>75	137(8.7)	49(17.7)			79(37.1)	18(45.0)	
Sex			0.120				0.878
Male	1187(75.1)	220(79.4)			109(51.2)	21(52.5)	
Female	394(24.9)	57(20.6)			104(48.8)	19(47.5)	
BMI(kg/m2)			0.636				0.198
<25	1275(80.6)	220(79.4)			35(51.5)	16(66.7)	
≥25	306(19.4)	57(20.6)			33(48.5)	8(33.3)	
ASA score			0.002				0.764
1	987(62.4)	152(54.9)			11(5.2)	1(2.5)	
2	541(34.2)	105(37.9)			48(22.5)	9(22.5)	
3-4	53(3.4)	20(7.2)			154(72.3)	30(75.0)	
Comorbidities			0.032				0.004
No	1101(69.6)	175(63.2)			142(66.7)	17(42.5)	
Yes	480(30.4)	102(36.8)			71(33.3)	23(57.5)	
Previous surgery			0.488				0.017
No	1234(78.1)	211(76.2)			157(73.7)	22(55.0)	
Yes	347(21.9)	66(23.8)			56(26.3)	18(45.0)	
Neoadjuvant therapy			0.446				0.007
No	1547(97.8)	269(97.1)			192(90.1)	30(75.0)	
Yes	34(2.2)	8(2.9)			21(9.9)	10(25.0)	
Tumor location			0.858				0.858
Upper	504(31.9)	89(32.1)			504(31.9)	89(32.1)	
Middle	389(24.6)	64(23.1)			389(24.6)	64(23.1)	
Total	688(43.5)	124(44.8)			688(43.5)	124(44.8)	
Tumor size(cm)			0.890				0.205
<4.6 cm	829(52.4)	144(52.0)			54(58.1)	16(72.70)	
≥4.6 cm	752(47.6)	133(48.0)			39(41.9)	6(27.3)	
Anastomosis approach			0.516				0.027
Intra-corporeal	82(5.2)	17(6.1)			176(82.6)	27(67.5)	
Extra-corporeal	1499(94.8)	260(93.9)			37(17.4)	13(32.5)	
Type of resection			0.932				0.798
Distal gastrectomy	687(43.5)	124(44.8)			145(68.1)	25(62.5)	
Total gastrectomy	859(54.3)	148(53.4)			56(26.3)	13(32.5)	
Proximal gastrectomy	34(2.2)	5(1.8)			10(4.7)	2(5.0)	
Pylorus-preserving gastrectomy	1(0.1)	0			2(0.19	0	
Type of reconstruction			0.923				0.630
B-I	581(36.7)	101(36.5)			0	0	
B-II	99(6.3)	22(7.9)			34(16.0)	3(7.5)	
Roux-en-Y GJ	7(0.4)	1(0.4)			111(52.1)	22(55.0)	
Roux-en-Y EI	869(54.3)	148(53.4)			56(26.3)	14(35.0)	
Esophagogastrostomy	34(2.2)	5(1.8)			10(4.7)	1(2.5)	
Gastrogastrostomy	1(0.1)	0			2(0.9)	0	
Type of lymphadenectomy			0.659				0.889
D1/ D1+	23(1.5)	5(1.8)			88(41.3)	17(42.5)	
D2	1558(98.5)	272(98.2)			125(58.7)	23(57.5)	
Combined resection			0.058				0.006
No	1419(89.8)	238(85.9)			178(83.6)	26(65.0)	
Yes	162(10.2)	39(14.1)			35(16.4)	14(35.0)	
Operation time (min)			0.151				0.028
<180	1167(73.8)	193(69.7)			120(56.3)	15(37.5)	
≥180	414(26.2)	84(30.3)			93(43.7)	25(62.5)	
Estimated blood loss (ml)			0.004				0.006
<75	1277(80.8)	203(73.3)			121(72.0)	18(48.6)	
≥75	304(19.2)	74(2.7)			47(28.0)	19(51.4)	
No. of retrieved LNs			0.102				0.086
<34	881(55.7)	169(61.0)			127(59.6)	18(45.0)	
≥34	700(44.3)	108(39.0)			86(40.4)	22(55.0)	
Histological			0.775				0.775
Differentiated	1115(70.5)	193(69.7)			1115(70.5)	193(69.7)	
Undifferentiated	466(29.5)	84(30.3)			466(29.5)	84(30.3)	
pT stage			0.672				0.046
T1	373(23.6)	65(23.5)			106(49.8)	20(50.0)	
T2	176(11.1)	24(8.7)			32(15.0)	3(7.5)	
T3	447(28.3)	85(30.7)			60(28.2)	9(22.5)	
T4	585(40.0)	103(37.2)			15(7.0)	8(20.0)	
pN stage			0.140				0.030
N0	570(36.1)	85(30.7)			132(62.0)	24(60.0)	
N1	224(14.2)	45(16.2)			33(15.5)	2(5.0)	
N2	266(16.8)	41(14.8)			24(11.3)	3(7.5)	
N3a	310(19.6)	70(25.3)			19(8.9)	10(25.0)	
N3b	211(13.3)	36(13.0)			5(2.3)	1(2.5)	
pTNM stage			0.683				0.188
IA	311(19.7)	54(19.5)		I	122(57.3)	21(52.5)	
IB	135(8.5)	17(6.1)		II	48(22.5)	6(15.0)	
IIA	162(10.2)	23(8.3)		III	43(20.2)	13(32.5)	
IIB	170(10.8)	36(13.0)					
IIIA	160(10.1)	29(10.5)					
IIIB	254(16.1)	45(16.2)					
IIIC	389(24.6)	73(26.4)					

**Table 5:** Multivariate analysis of the morbidity for eastern and western laparoscopy gastrectomy

Variable	B	S.E	Wals	P	OR	95%CI
**Eastern centers**						
Age	0.773	0.173	20.064	0.000	2.166	1.545-3.038
ASA score	0.369	0.155	5.660	0.017	1.446	1.067-1.960
Comorbidities	0.198	0.203	0.953	0.329	1.219	0.819-1.814
Combined resection	-0.343	0.195	3.092	0.079	0.710	0.484-1.040
Estimated blood loss	0.439	0.152	8.353	0.004	1.551	1.152-2.089
Constant	-3.842	0.532	52.222	0.000	0.021	
**Western centers**						
Comorbidities	-0.244	0.592	0.169	0.681	0.784	0.245-2.502
Previous surgery	-0.617	0.686	0.810	0.368	0.539	0.141-2.069
Neoadjuvant therapy	-1.471	0.584	6.345	0.012	0.230	0.073-0.722
Anastomosis approach	-0.583	0.540	1.167	0.280	0.558	0.194-1.608
Combined resection	-0.179	0.583	0.094	0.759	0.836	0.267-2.623
Operation time	-0.142	0.685	0.043	0.836	0.867	0.227-3.322
Estimated blood loss	-0.677	0.650	1.086	0.297	0.508	0.142-1.816
No. of retrieved LNs	-0.933	0.459	4.136	0.042	0.394	0.160-0.967
pT stage			7.792	0.051		
pT1 vs pT2	-0.866	0.781	1.227	0.268	0.421	0.091-1.946
pT1 vs pT3	-1.926	0.981	3.855	0.050	0.146	0.021-0.997
pT1 vs pT4	-1.931	0.796	5.889	0.015	0.145	0.030-0.690
pN stage			5.827	0.212		
pN0 vs pN1	0.102	1.736	0.003	0.953	1.107	0.037-33.265
pN0 vs pN2	-1.544	1.861	0.688	0.407	0.214	0.006-8.204
pN0 vs pN3a	-0.987	1.817	0.295	0.587	0.373	0.011-13.116
pN0 vs pN3b	0.470	1.717	0.295	0.784	1.600	0.055-46.320
Constant	3.019	1.791	2.843	0.092	20.481	
